# Do caregiver cooking skills boost adolescent resilience and prosocial behavior? Results from a population-based longitudinal study in Japan

**DOI:** 10.1186/s40359-026-04658-4

**Published:** 2026-05-01

**Authors:** Yukako Tani, Aya Isumi, Takeo Fujiwara

**Affiliations:** 1 Department of Public Health, Institute of Science Tokyo, 1-5-45, Yushima, Bunkyo-ku, Tokyo 113-8510 Japan; 2 Department of Health Policy, Institute of Science Tokyo, 1-5-45, Yushima, Bunkyo-ku, Tokyo 113-8510 Japan

**Keywords:** Cooking skills, Adolescent, Resilience, Prosocial behavior, Japan

## Abstract

**Purpose:**

Promoting positive mental health in adolescence is important for life-course well-being. We sought to examine whether caregivers’ cooking skills are associated with the promotion of adolescents’ resilience and prosocial behavior in Japan.

**Methods:**

We used longitudinal data from 2018 to 2020 from the Adachi Child Health Impact of Living Difficulty (A-CHILD) study. The baseline survey was administered to all fourth-grade elementary school students (9–10 years old) and their caregivers, and a follow-up survey was administered 2 years later (*n* = 3,641, follow-up rate = 87%). Caregiver cooking skills were assessed at baseline using a cooking skills scale modified for use in Japan. Child resilience and prosocial behavior in fourth and sixth grade were assessed by caregivers using the Children’s Resilient Coping Scale and the Strength and Difficulties Questionnaire, respectively, and scores were rescaled to a 0–100 metric to facilitate interpretation. Multivariable linear regression models were used to examine associations between caregiver cooking skills and child outcomes after adjustment for potential confounders. Mediation analyses estimated indirect effects through food-related household routines, caregiver-child interactions, and family cohesion.

**Results:**

In multivariable linear regression analyses, higher baseline caregiver cooking skills were associated with higher resilience and prosocial behavior scores at follow-up. Compared with children in the lowest quartile, those in the highest quartile had resilience scores that were 8.75 points higher (95% CI: 7.38 to 10.1) and prosocial behavior scores that were 9.51 points higher (95% CI: 7.68 to 11.3) in the model adjusted for potential confounders. These associations were partially mediated by food-related household routines, caregiver-child interactions, and family cohesion.

**Conclusions:**

For early adolescents in Japan, caregivers’ cooking skills were associated with children’s positive mental health. An educational program that allows caregivers to learn cooking skills may be important in promoting adolescent positive mental health.

**Supplementary Information:**

The online version contains supplementary material available at 10.1186/s40359-026-04658-4.

## Introduction

Promoting resilience and prosocial behaviors during adolescence is important for protecting them from mental health problems throughout their lives [1, 2]. This is because adolescence is the time when most mental health problems begin [3]. The peak age of onset of mental disorders is 15 years, with 75% of cases occurring by age 25 [4, 5]. Adolescent mental health problems are the greatest cause of loss of human potential and productivity throughout life [6–8]. Resilience refers to the capacity for, or outcome of, successful adaptation despite challenging or threatening circumstances [9, 10]. A recent systematic review of adolescents and young adults showed that resilience is inversely associated with mental health problems (depression, anxiety, etc.) and positively associated with positive mental health indicators (well-being, life satisfaction, etc.) [1]. Prosocial behaviors refer to positive social actions toward others, and these actions are intended to promote the welfare of others [11]. Recent review of adolescents showed a positive association between prosocial behavior and mental health [2]. Therefore, developing resilience and prosocial behavior by early adolescence is important for all children’s mental health and well-being later in life .

Adolescent resilience and prosocial behavior are influenced by both extrapersonal and interpersonal factors [12–15]. At the intrapersonal factors, empathy and self-regulation are consistently associated with higher resilience and prosocial behavior [12–15]. At the extrapersonal factors, family cohesion, caregiver positive involvement, teaching children self-care skills, and consistent household routines are known home environmental factors that can facilitate child resilience [12, 13]. Similarly, supportive parenting, family cohesion, and parental socialization practices have been suggested to foster children’s prosocial behavior [14]. In addition, shared family routines, such as family meals, provide important contexts for repeated social interaction, emotional support, and behavioral modeling [16]. Therefore, the home environment plays a central role in the development of child resilience and prosocial behavior. In the home environment, caregiver cooking skills may represent a potential facilitating factor for children’s resilience and prosocial behavior.

The association between caregivers’ cooking skills and children’s resilience can be understood through an integration of social learning theory, family systems theory, and ecological models. From a social learning perspective [17], everyday cooking and mealtime contexts provide opportunities for modeling adaptive behaviors such as cooperation, problem-solving, and emotional regulation, which children acquire through observation, imitation, and reinforcement within parent–child interactions. Cooking is the most representative household routine, and better cooking skills of caregivers can lead to a stable food supply for children and ensure the quality of their diet [18]. Furthermore, if caregivers have sufficient cooking skills, they can teach their children to cook. Mothers are the main source for teaching cooking skills, and those who learned cooking skills from their mothers had higher cooking skills and ate healthier than those who learned from other sources (friends, school, other family members, etc.) [19]. In a U.S. study of adolescents, the more often they were engaged in meal preparation and shopping, the more frequently they consumed fruits/vegetables, and the higher the quality of their diet [20]. A study of Japanese adolescents reported that when caregivers have poor cooking skills, they cook less frequently at home and their children consume vegetables less frequently [18]. Thus, caregiver cooking skills can ensure food-related household routines and provide opportunities for children to acquire meal-related practical skills. From the perspective of family systems theory [21], cooking and shared meals function as central organizing practices that structure communication, role allocation, and emotional bonding within the family; thus, higher caregiver cooking skills may strengthen family cohesion and create a stable and supportive environment conducive to resilience. Furthermore, within Bronfenbrenner’s Ecological Systems Theory [22], food-related household routines represent repeated and enduring processes within the microsystem that shape children’s daily experiences and support the development of self-regulation and coping capacities. Consistent household routines are essential for children to establish feelings of safety, learn to care for themselves, and form the basis for reducing toxic stress [13]. These processes, in turn, support the development of self-regulation and coping capacities.

The association between caregivers’ cooking skills and children’s prosocial behavior can be understood through an integration of multiple theoretical frameworks. From the perspective of social learning theory [17], everyday cooking and mealtime contexts provide opportunities for modeling prosocial behaviors such as cooperation and consideration, through which children acquire these behaviors via observation, imitation, and social reinforcement within caregiver–child interactions. In addition, family systems theory conceptualizes cooking and shared meals as central organizing activities that structure roles, communication, and relationships within the family; thus, higher caregiver cooking skills may enhance family cohesion and create a stable emotional climate that supports children’s social development. Furthermore, within Bronfenbrenner’s Ecological Systems Theory, food-related household routines can be viewed as repeated and enduring processes within the microsystem that provide consistent contexts for socialization and the internalization of social norms.

Caregiver cooking skills may influence family cohesion and caregiver-child interactions through several specific mechanisms. First, higher cooking skills may facilitate more frequent shared meals, which provide structured opportunities for caregiver–child communication [16]. Second, meal preparation may create teaching moments in which caregivers engage children in collaborative activities, fostering interaction and mutual support, Third, preparing meals for the family may function as a demonstration of care, strengthening emotional bonds. Additionally, greater time spent together during meal-related activities may further enhance family cohesion. A study of elementary school students and their caregivers in Japan showed that frequent home cooking is associated with higher child resilience, and this association was mediated by parent-child interactions such as talking about school life [23]. Thus, the caregiver’s cooking skills can provide opportunities for interaction with their child, foster family cohesion, and potentially facilitate the child’s resilience and prosocial behavior.

Caregiver cooking skills are conceptually distinct from the frequency of home cooking or family meals. Whereas frequency of home cooking or family meals reflects how often food is prepared or shared, cooking skills refer to the practical abilities and confidence required to plan, prepare, and adapt meals in ways that meet household needs [24, 25]. Thus, caregivers with stronger cooking skills may be better able to provide nutritious meals [26], involve children in meal preparation, and maintain food-related routines even under time or resource constraints. Conversely, frequent home cooking does not necessarily imply high skill, and high skill does not always translate into frequent cooking. Examining cooking skills specifically may therefore identify a more modifiable and actionable family resource than meal frequency alone.

The Japanese context may be particularly relevant for examining the association between caregivers’ cooking skills and children’s resilience and prosocial behavior. In Japan, meals are traditionally viewed as an important setting for family connection and socialization, and the emotional significance of home-prepared meals is reflected in the cultural concept of *ofukurono-aji* (“the taste of home cooking”) [27]. A survey of Japanese children aged 10–14 years found that they spent only 2–3 h with their parents on weekdays, and meals were the main activity during which they spent time together [28]. Among parents of Japanese fourth-grade children, approximately 70% of mothers were employed; among them, only about 70% returned home by 6:00 p.m [23]., whereas fewer than 10% of fathers returned home by that time, as confirmed in the present study. Therefore, within the Japanese context, caregivers’ cooking skills may be particularly important during early adolescence as a means of supporting family connection and promoting children’s positive development.

Although caregiver cooking skills may influence children’s developmental outcomes, the association may also reflect broader family characteristics such as household organization, parenting style, and socioeconomic resources. Selection processes may also contribute if health-oriented families are more likely to facilitate cooking skills. Reverse causation is another possibility, as families with better-adjusted children may have a greater capacity to invest in home cooking. To reduce these concerns, the present study assessed caregiver cooking skills in fourth grade and examined children’s resilience and prosocial behavior two years later, in sixth grade, while accounting for family background factors such as parental education, employment status, return-home time, and household income.

The purpose of this study was first to examine whether caregivers’ cooking skills are associated with children’s resilience and prosocial behavior using a longitudinal study of adolescents and their caregivers in Japan. Second, we aimed to examine whether the associations between caregiver cooking skills and children’s resilience/prosocial behaviors were mediated by family cohesion, caregiver caregiver-child interactions, and food-related household routines. We hypothesized that higher caregiver cooking skills would be associated with higher levels of resilience and prosocial behavior at follow-up. We further hypothesized that these associations would be partially mediated by family cohesion, caregiver-child interactions, and food-related household routines, with family cohesion and caregiver-child interactions expected to play particularly important roles as proximal relational processes in adolescent development.

## Materials and methods

### Study design and participants

This study used data from the Adachi Child Health Impact of Living Difficulty (A-CHILD) project, established in 2015 to assess health determinants among children in Adachi, Tokyo, Japan [29]. The study used longitudinal data conducted on fourth-grade students (ages 9–10) in October 2018 and subsequently followed through sixth-grade students (ages 11–12) in October 2020 (follow-up period: 2 years). The survey was administered to 5,311 fourth-grade elementary school students and their caregivers in all 69 public elementary schools in Adachi City, Tokyo, using self-report questionnaires with anonymous unique IDs. A total of 4,605 pairs responded to the questionnaires (response rate: 86.7%). Of these, 4,290 pairs provided informed consent and completed all surveys at baseline. 87% of baseline participant pairs completed the follow-up self-report questionnaires in 2020 (*n* = 3,733). For the analysis, 3,641 pairs were included, excluding those who had incomplete information on child sex (*n* = 7) and those who did not complete the questions related to child resilience (*n* = 7) and prosocial behaviors (*n* = 78). A flow chart of the participants is presented in Supplementary Fig. 1. The A-CHILD protocol and use of the data for this study were approved by the Ethics Committee at the Tokyo Medical and Dental University (No. M2016-284).

### Child resilience and prosocial behaviors

Resilience was assessed using the Children’s Resilient Coping Scale (CRCS) at baseline and at follow-up through a caregiver questionnaire. This scale was developed by Japanese experts to suit the Japanese context and has high internal consistency (Cronbach’s alpha = 0.80) and sufficient validity [30]. CRCS consists of the following eight items: (1) speaks positively about their future; (2) tries to do their best; (3) able to tolerate teasing or mean comments well; (4) knows how to properly greet others; (5) able to get ready for school, study, and do their chores without directions; (6) seeks appropriate advice when necessary; (7) able to give up things they want or do things that they do not like to do for better future outcomes; and (8) able to ask questions to learn about what they do not understand. Caregivers rated their child’s resilience on a 5-point Likert scale from 0 (never) to 4 (very frequently). The total score of the eight items was rescaled to range from 0 to 100, with higher scores indicating higher resilience. The Cronbach’s α of CRCS for the study sample was 0.86.

Prosocial behavior was assessed using the Strengths and Difficulties Questionnaire (SDQ) at baseline and at follow-up through a caregiver questionnaire [31]. Caregivers rated their child’s prosocial behaviors on a 3-point Likert scale from 0 (not true) to 2 (certainly true). A total score of five items was calculated and rescaled to range from 0 to 100 to aid in the interpretation of coefficients in the statistical analysis, following previous work [30, 32]. A higher prosocial behavior score corresponded to a higher level of prosocial behavior. The Cronbach’s α of prosocial behaviors for the study sample was 0.71.

### Caregiver cooking skills

Caregiver cooking skills were assessed at baseline using a modified cooking skills scale developed to take into account basic Japanese cooking methods and typical meals [18, 33]. The scale consists of five items: (1) able to peel fruits and vegetables; (2) able to make stir-fried meat and vegetables; (3) able to make miso soup; (4) able to make stewed dishes; and (5) like to cook. Items 1–4 were derived from basic Japanese cooking methods, and item 5 was added as an indicator of cooking skill, as a study on the life course trajectory of cooking skills reported that a liking for cooking is a characteristic of those who maintain high cooking skills [34]. Caregivers were asked to rate their cooking skills on a 6-point Likert scale from not at all disagree (= 1 point) to very much agree (= 6 points). Higher scores indicated that caregivers had high confidence in their cooking skills. The Cronbach’s α of the cooking skills scale for the study sample was 0.78. The means of these five items were calculated and divided into quartiles for analysis.

### Potential mediators

Potential mediators were assessed at the baseline survey using caregiver-completed questionnaires, except for the child’s frequency of breakfast intake and eating dinner with caregivers and children, which were self-reported by the child. We considered food-related household routines, caregiver-child interactions, and family cohesion as possible mediators (Supplementary Fig. 2). Food-related household routines included children’s eating behaviors (frequency of vegetable intake, frequency of breakfast intake, eating dinner with caregivers and children) [23], frequency of caregivers’ home cooking, frequency of cooking with caregiver and child, and frequency of going out with caregiver and child were included because it was hypothesized that higher caregivers’ cooking skills can promote grocery shopping and cooking with the child.

Caregiver-child interactions included the frequency of talking with their children about school life, news, and TV shows because a previous study has shown that more frequent home cooking was associated with more conversation between caregivers and children [23]. Family cohesion was assessed by seven items from the Family Social Capital Scale [35]. These items are related to cognitive aspects (e.g., perception of working well as a family, perception of importance of following family’s rule, reliability of family members for support and help, etc.) modified to fit Japanese family styles [36]. Caregivers were asked to rate their family cognitive social capital on a 5-point Likert scale from not at all disagree (= 0 point) to very much agree (= 4 points). The mean score of the seven items is calculated, and a higher score indicates higher family social capital. The Cronbach’s α of the family social capital scale for the study sample was 0.88, and factor analysis confirmed that it was a single factor.

### Covariates

Covariates were also obtained from the baseline caregiver questionnaire. Information on maternal and paternal characteristics was provided by the responding caregiver, including when the respondent was a grandparent or other caregiver. Caregiver status included the responding caregiver and his/her mental health, parental age, educational attainment (low: middle school, high school dropout, high school; middle: vocational school, some college, college dropout; high: college or higher), and employment/daily time of returning home. The mental health of the responding caregivers was assessed using the Kessler 6 scale, with higher scores indicating more frequent psychological distress problems [37, 38]. Parental working and returning home time was classified into five groups (working, returning home before 18:00; working, returning home between 18:00 and 20:00; working, returning home after 20:00; working, time irregular, not working) [23]. Participants with missing data on the covariates were included in the analysis.

### Statistical analysis

First, the baseline characteristics of children and their caregivers were stratified by quartile of caregiver cooking skills, and the differences in characteristics were tested using Chi-square tests or ANOVA. Second, to examine the association between caregiver cooking skills and child resilience/prosocial behaviors, a multivariate linear regression model was performed. In Model 1, baseline variables that were associated with caregiver cooking skills in the analyses described above were adjusted for potential confounders. In Model 2, baseline child resilience/prosocial behaviors were adjusted for in addition to Model 1. P for trend was calculated by entering cooking skill quartiles as an ordinal variable in the regression models. To facilitate interpretation of the magnitude of associations, standardized effect sizes (Cohen’s d) were calculated by dividing the adjusted regression coefficients by the standard deviation of each outcome measure. We conducted several sensitivity analyses to assess the robustness of the findings. First, analyses were stratified by household income level to examine potential differences by socioeconomic status. Second, analyses were stratified by child sex. Third, because information on the primary household cook was unavailable, we repeated the analyses restricted to households in which the respondent was the mother, as mothers are most commonly responsible for meal preparation in Japan. Fourth, we examined alternative covariate adjustment models by sequentially adding child, household, and caregiver characteristics. To examine whether the associations between caregiver cooking skills and child outcomes differed according to children’s initial levels of resilience or prosocial behavior, we additionally tested interaction terms between caregiver cooking skills and baseline outcome scores. A mediation analysis was conducted to determine the proportion of the association between caregiver cooking skill and child resilience/prosocial behaviors that was mediated by the potential mediators. Possible mediating factors included food-related household routines, caregiver-child interactions, and family cohesion that were associated with caregiver cooking skills in the first analysis. Mediation analyses were performed using the paramed package in Stata [39]. We conducted mediation analyses by examining each of the eight mediators separately across the three domains. No exposure–mediator interaction terms were included in the analyses. The proportion mediated was calculated as the indirect effect divided by the total effect, expressed as a percentage. Confidence intervals were estimated using nonparametric bootstrapping with 1,000 replications. All models were adjusted for potential confounders. These analyses were intended to identify potential explanatory pathways rather than to support causal mediation, as assumptions of no unmeasured confounding cannot be fully guaranteed in this observational study. All analyses were conducted using Stata statistical software Macro Package version 17 (Stata Corporation, TX, USA).

## Results

The characteristics of the participants at baseline are shown in Table 1. Almost half of the children were girls, 40% ate vegetables at least twice a day, and 90% of the children had breakfast every day and ate dinner with their caregivers. 92% of respondents to the caregiver survey were mothers, 7% were fathers, and 1% were other caregivers. Caregiver cooking skills differed by paternal educational attainment and parental employment status. Among the potential mediators of food-related household routines, the frequency of the child’s eating breakfast and whether or not to eat dinner with caregivers and children were not related to the level of caregiver cooking skills. The other potential mediators of food-related household routines (frequency of child’s vegetable intake, frequency of caregiver’s home cooking, frequency of cooking with caregiver and child, and frequency of going out with caregiver and child) were more frequent when the caregiver’s cooking skills were high. Potential mediators other than food-related household routines, such as caregiver-child interactions and family cohesion, the higher the caregiver’s cooking skills, the more often the caregiver and child talked, and the higher the family social capital.

**Table 1:** Baseline characteristics of children and caregivers in Japanese school children (*n* = 3,641)

	Total		Caregiver cooking skills				*p*-value^a^
			Q1 (low)	Q2	Q3	Q4 (high)	
			*n* = 1160	*n* = 893	*n* = 696	*n* = 892	
	N or Mean	% or SD	% or mean	% or mean	% or mean	% or mean	
Child’s status							
Sex							
Boy	1821	50.0	51.2	53.3	47.6	47.1	0.03
Girl	1820	50.0	48.8	46.7	52.4	52.9	
Eating behaviors							
Frequency of child’s vegetable intake							
Twice or more /day	1508	41.4	31.6	36.2	50.6	52.4	< 0.001
Once/day	1765	48.5	50.3	55.0	45.1	42.2	
Less than 3 times/weeks	362	9.9	17.9	8.6	4.3	5.3	
Missing	6	0.2	0.2	0.2	0.0	0.2	
Frequency of child’s breakfast intake							
Everyday	3293	90.4	89.5	90.0	92.7	90.4	0.44
Often	248	6.8	7.5	7.6	5.0	6.5	
Rarely/never	66	1.8	1.9	1.8	1.3	2.1	
Missing	34	0.9	1.1	0.6	1.0	1.0	
Eating dinner with caregivers and children							
No (non-family meal)	260	7.1	7.7	7.7	6.5	6.4	0.53
Yes	3381	92.9	92.3	92.3	93.5	93.6	
Positive mental health							
Resilience (CRCS score: 0-100)	69.6	16.3	64.0	69.3	72.8	74.7	< 0.001
Prosocial behaviors (SDQ score: 0-100)	67.3	20.8	62.4	66.4	69.4	72.9	< 0.001
Household status							
Cohabitation status							
Parents	2931	80.5	78.8	83.8	83.0	77.5	0.007
Parents and grandparent (s)	278	7.6	7.9	7.3	7.5	7.7	
Single parent	356	8.0	8.6	6.4	5.9	10.7	
Single parent and grandparent (s)	39	2.8	3.7	1.7	2.7	2.8	
Other	37	1.0	0.9	0.9	0.9	1.3	
Other children in the household (sibling)							
No	724	19.9	22.3	17.6	19.4	19.4	0.06
Yes	2917	80.1	77.7	82.4	80.6	80.6	
Household income (million yen)							
< 3.00	354	9.7	9.6	9.3	7.8	11.9	0.003
3.00–5.99	1083	29.7	32.9	31.8	27.4	25.3	
6.00-9.99	1256	34.5	31.5	34.4	39.1	35.0	
≥ 10.0	455	12.5	12.3	12.0	12.9	12.9	
Missing	493	13.5	13.7	12.5	12.8	14.9	
Caregiver’s status							
Respondent caregiver							
Mother	3332	91.5	86.4	96.1	96.0	90.1	< 0.001
Father	269	7.4	12.5	3.4	3.0	8.2	
Other	40	1.1	1.1	0.6	1.0	1.7	
Respondent’s K6							
< 5	2406	66.1	57.4	66.4	72.8	71.7	< 0.001
≥ 5	1126	30.9	39.5	30.1	25.0	25.2	
Missing	109	3.0	3.1	3.5	2.2	3.0	
Mother’s age (years)							
< 35	369	10.1	10.3	9.2	10.5	10.7	0.17
35–44	2208	60.6	58.8	61.1	62.8	60.9	
≥ 45	965	26.5	27.4	27.8	25.0	25.2	
Missing	99	2.7	3.5	1.9	1.7	3.3	
Father’s age (years)							
< 35	164	4.5	5.0	4.5	4.3	4.0	0.04
35–44	1735	47.7	46.3	49.9	50.0	45.3	
≥ 45	1357	37.3	37.7	37.4	36.5	37.2	
Missing	385	10.6	11.0	8.2	9.2	13.5	
Mother’s education							
Low	944	25.9	28.1	25.3	24.9	24.6	0.26
Middle	1233	33.9	31.6	34.7	35.9	34.4	
High	572	15.7	15.3	17.0	16.2	14.6	
Other/missing	892	24.5	25.1	23.0	23.0	26.5	
Father’s education							
Low	974	26.8	28.1	26.0	27.0	25.6	0.002
Middle	580	15.9	15.2	18.8	13.6	15.8	
High	1069	29.4	27.5	30.5	33.9	27.1	
Other/missing	1018	28.0	29.2	24.7	25.4	31.5	
Mother’s employment and go home time							
Employed and go home before 18:00	1698	46.6	45.6	48.4	48.6	44.7	0.002
Employed and go home from 18:00–20:00	531	14.6	14.8	15.2	13.5	14.5	
Employed and go home after 20:00	123	3.4	3.9	2.8	2.7	3.8	
Employed and go home irregularly	113	3.1	3	2.2	3.3	3.9	
Not employed	793	21.8	20.9	23	24.6	19.6	
Missing	383	10.5	11.8	8.4	7.3	13.5	
Father’s employment and go home time							
Employed and go home before 18:00	276	7.6	9.2	6.5	6	7.7	0.004
Employed and go home from 18:00–20:00	867	23.8	24.8	24.4	23.3	22.3	
Employed and go home after 20:00	1367	37.5	35.1	40.2	41.5	35	
Employed and go home irregularly	582	16	15.5	16.9	15.5	16	
Not employed	32	0.9	0.9	0.7	0.9	1.1	
Missing	517	14.2	14.5	11.3	12.8	17.8	
Food-related household routines							
Frequency of caregiver’s home cooking							
≥ 6 days/week	3165	86.9	78.4	88.4	92.2	92.4	< 0.001
4–5 days/week	383	10.5	16.2	10.0	6.8	6.6	
≤ 3 days/week (low frequency)	83	2.3	5.0	1.5	0.7	0.8	
Missing	10	0.3	0.3	0.2	0.3	0.2	
Frequency of cooking with caregiver and child (n/weeks)	0.7	1.1	0.7	1.1	0.7	1.1	< 0.001
Frequency of going out with caregiver and child (n/weeks)	2.1	1.7	1.9	2.0	2.1	2.3	< 0.001
Caregiver-child interactions							
Frequency of talking about school life with caregiver and child (n/weeks)	5.7	2.1	5.2	5.9	6.0	6.0	< 0.001
Frequency of talking about news with caregiver and child (n/weeks)	1.9	2.2	1.5	1.9	2.1	2.4	< 0.001
Frequency of talking about TV shows with caregiver and child (n/weeks)	3.8	2.5	3.5	3.8	4.1	4.0	< 0.001
Family cohesion							
Family social capital score (0–4)	3.56	0.5	3.4	3.6	3.6	3.7	< 0.001

*K6*  Kesseler 6, *SD*  Standard Deviation

^a^Chi-squared test for categorical variables and ANOVA for continuous variables were used to examine statistical significance

The resilience scores and prosocial behavior scores of children at baseline (grade 4) and at follow-up (grade 6) are shown in Supplementary Table 1. Supplementary Table 2 has also been added, presenting child outcomes at follow-up across caregiver cooking skill quartiles. The average child resilience score was 69.6 (SD = 16.3) at baseline and 71.7 (SD = 16.3) at follow-up (grade 6) (difference = 2.05, 95% CI, 1.60 to 2.51). The average score of child prosocial behaviors was 67.3 (SD = 20.8) at baseline, but this slightly dropped to 65.5 (SD = 21.3) at follow-up (difference = − 1.72, 95% CI, − 2.37 to − 1.06).

The associations between caregiver cooking skills and child resilience/prosocial behaviors are shown in Table 2. Compared with children with the lowest quartile (Q1) level of caregiver cooking skills, those with a higher level of caregiver cooking skills at baseline had higher resilience at follow-up (coefficient for Q4 = 8.75, 95% CI: 7.38 to 10.1, coefficient for Q3 = 5.89, 95% CI: 4.40 to 7.37, coefficient for Q2 = 4.06, 95% CI: 2.69 to 5.44), after adjusting for potential confounders (Model 1). These corresponded to standardized effect sizes (Cohen’s d) of 0.54 for Q4, 0.36 for Q3, and 0.25 for Q2, based on the overall standard deviation of the resilience score. This association was significant after adjusting for baseline child resilience (Model 2). For prosocial behaviors, children with a higher level of caregiver cooking skills at baseline had higher prosocial behaviors at follow-up (coefficient for Q4 = 9.51, 95% CI: 7.68 to 11.3, coefficient for Q3 = 6.31, 95% CI: 4.32 to 8.29, coefficient for Q2 = 4.72, 95% CI: 2.89 to 6.56), compared with those with the Q1 of caregiver cooking skills (Model 1). These corresponded to standardized effect sizes (Cohen’s d) of 0.45 for Q4, 0.30 for Q3, and 0.22 for Q2, based on the overall standard deviation of the resilience score. This association was significant after adjusting for baseline child prosocial behaviors (Model 2). A significant positive trend across cooking skill quartiles was observed for both resilience and prosocial behavior in all models (all p for trend < 0.001). No statistically significant interactions were observed between caregiver cooking skills and baseline resilience or prosocial behavior scores (all p for interaction > 0.05), indicating that the associations did not differ by children’s initial outcome levels (Supplementary Tables 3 and 4). Sensitivity analyses showed similar patterns of association when stratified by household income and child sex (Supplementary Tables 5 and 6). Results were also materially unchanged when analyses were restricted to mother respondents and when alternative covariate adjustment models were applied (Supplementary Tables 7 and 8).

**Table 2:** Associations of baseline caregiver cooking skills with child resilience and prosocial behavior at 2-year follow-up: multivariable linear regression analyses (*n* = 3,641)

Caregiver’s cooking skills	Unadjusted mean score at follow-up (SD)	Crude		Model 1		Model 2	
		coefficient (95%CI)	Cohen’s d	coefficient (95%CI)	Cohen’s d	coefficient (95%CI)	Cohen’s d
Resilience (CRCS score)							
Q1 (Low)	66.7 (16.9)	ref	ref	ref	ref	ref	ref
Q2	71.5 (15.5)	**4.85 (3.46 to 6.23)**	**0.30**	**4.06 (2.69 to 5.44)**	**0.25**	**2.65 (1.45 to 3.85)**	**0.16**
Q3	74.0 (14.7)	**7.33 (5.84 to 8.82)**	**0.45**	**5.89 (4.40 to 7.37)**	**0.36**	**2.85 (1.54 to 4.16)**	**0.17**
Q4 (High)	76.5 (15.6)	**9.77 (8.39 to 11.2)**	**0.60**	**8.75 (7.38 to 10.1)**	**0.54**	**4.73 (3.52 to 5.95)**	**0.29**
P for trend		< 0.001		< 0.001		< 0.001	
Prosocial behavior (SDQ score)							
Q1 (Low)	60.5 (21.6)	ref	ref	ref	ref	ref	ref
Q2	65.3 (20.9)	**4.76 (2.93 to 6.6)**	**0.22**	**4.72 (2.89 to 6.56)**	**0.22**	**3.25 (1.57 to 4.94)**	**0.15**
Q3	67.4 (20.2)	**6.90 (4.93 to 8.9)**	**0.32**	**6.31 (4.32 to 8.29)**	**0.30**	**4.01 (2.18 to 5.83)**	**0.19**
Q4 (High)	70.8 (20.8)	**10.3 (8.45 to 12.1)**	**0.48**	**9.51 (7.68 to 11.3)**	**0.45**	**6.61 (4.91 to 8.30)**	**0.31**
P for trend		< 0.001		< 0.001		< 0.001	

*CI*  Confidence interval, *CRCS*  Children’s Resilient Coping Scale, *SD*  Standard Deviation, *SDQ*  Strengths and Difficulties Questionnaire

Coefficients represent mean differences in follow-up resilience or prosocial behavior scores compared with Q1 (lowest caregiver cooking skills), estimated using linear regression models

Boldface indicates statistical significance (p<0.05)

Outcome scores range from 0 to 100, with higher scores indicating higher levels of resilience or prosocial behavior

P for trend was estimated by modeling cooking skill quartiles as an ordinal variable (1–4)

Cohen’s d represents standardized mean differences and was calculated by dividing the regression coefficients by the standard deviation of the corresponding outcome in the analytic sample

Model 1: Adjusted for child’s sex, cohabitation status, household income, respondent, respondent’s K6, mother’s employment status, father’s age, education, and employment status

Model 2: Model 1 + adjusted for baseline child resilience/prosocial behaviors

Table 3 displays the mediation results for the hypothesized mediators. We found evidence of mediation between the level of caregiver cooking skills and child’s resilience through food-related household routines, such as frequency of child’s vegetable intake (14.3%, *P* < 0.001), caregiver-child interactions, such as frequency of talking about school life with child (12.6%, *P* < 0.001), and family cohesion (22.4%, *P* < 0.001). For the association with child’s prosocial behaviors, we observed evidence of mediation through food-related household routines, such as frequency of cooking with caregiver and child (11.0%, *P* < 0.001), caregiver-child interactions, such as frequency of talking about school life with child (9.7%, *P* < 0.001), and family cohesion (21.7%, *P* < 0.001).

**Table 3:** Mediation analyses between caregivers’ cooking skills and children’s resilience/prosocial behavior

	Estimated Indirect Effect		Proportion Mediated, %
Mediator	coefficient (95%CI)	*P* value	
Resilience (CRCS Total score)			
Food-related household routines			
Frequency of child’s vegetable intake (n/weeks)	0.42 (0.27 to 0.58)	< 0.001	14.3
Frequency of caregiver’s home cooking (n/weeks)	0.12 (0.03 to 0.21)	0.007	4.2
Frequency of cooking with caregiver and child (n/weeks)	0.16 (0.04 to 0.28)	0.01	5.7
Frequency of going out with caregiver and child (n/weeks)	0.14 (0.06 to 0.22)	< 0.001	5.0
Caregiver-child interactions			
Frequency of talking about school life with caregiver and child (n/weeks)	0.36 (0.24 to 0.48)	< 0.001	12.6
Frequency of talking about news with caregiver and child (n/weeks)	0.50 (0.35 to 0.65)	< 0.001	16.3
Frequency of talking about TV shows with caregiver and child (n/weeks)	0.12 (0.05 to 0.20)	< 0.001	4.4
Family cohesion			
Family social capital (score)	0.63 (0.47 to 0.78)	< 0.001	22.4
Prosocial behavior (SDQ score)			
Food-related household routines			
Frequency of child’s vegetable intake (n/weeks)	0.16 (-0.03 to 0.35)	0.10	
Frequency of caregiver’s home cooking (n/weeks)	-0.02 (-0.13 to 0.10)	0.75	
Frequency of cooking with caregiver and child (n/weeks)	0.35 (0.18 to 0.53)	< 0.001	11.0
Frequency of going out with caregiver and child (n/weeks)	0.16 (0.05 to 0.26)	0.003	5.1
Caregiver-child interactions			
Frequency of talking about school life with caregiver and child (n/weeks)	0.30 (0.17 to 0.44)	< 0.001	9.7
Frequency of talking about news with caregiver and child (n/weeks)	0.38 (0.22 to 0.55)	< 0.001	11.4
Frequency of talking about TV shows with caregiver and child (n/weeks)	0.10 (0.02 to 0.19)	0.02	3.4
Family cohesion			
Family social capital (score)	0.71 (0.52 to 0.91)	< 0.001	21.7

*CI*  Confidence interval, *CRCS*  Children’s Resilient Coping Scale, *SDQ*  Strengths and Difficulties Questionnaire

Models were adjusted for child’s sex, cohabitation status, household income, respondent, respondent’s K6, mother’s employment status, father’s age, education, and employment status

Each mediator was examined in a separate model

Confidence intervals were estimated using nonparametric bootstrapping with 1,000 replications

Proportion mediated was calculated as the indirect effect divided by the total effect × 100

## Discussion

This longitudinal population-based study among adolescents and their caregivers in Japan found that a higher level of caregiver cooking skills when children were 9–10 years old was associated with higher resilience and prosocial behavior at ages 11–12. Household food-related routines, caregiver-child interactions, and family social capital mediated these associations.

To our knowledge, no established minimally important difference exists for the CRCS or the SDQ prosocial behavior score. However, previous methodological research suggests that approximately one-half of a standard deviation may represent a meaningful difference across a wide range of outcomes [40]. In this context, the observed associations in Model 1 (Q4 vs. Q1: 0.54 SD for resilience and 0.45 SD for prosocial behavior; Table 2) may be considered meaningful. Compared with other observational studies, the associations observed in the present study appear substantial. Children whose caregivers were in the highest quartile (Q4) of cooking skills had resilience scores that were 8.8 points higher than those in the lowest quartile (Q1). In previous studies using the same resilience scale, greater parental social network diversity was associated with only 0.75–1.0 point higher resilience scores [41], while two or more maternal adverse childhood experiences were associated with 2.8 points lower resilience scores [42]. For prosocial behavior, children in Q4 had scores that were 9.5 points higher than those in Q1. By comparison, greater parental social network diversity was associated with approximately a 1-point higher prosocial behavior score [41], and high parental social capital (tertile 3 vs. tertile 1) was associated with approximately a 3-point higher score [43]. 

When baseline resilience and prosocial behavior were included in the models (model 2 in Table 2), the standardized effect sizes were attenuated by approximately 30–40%. This pattern suggests that part of the association between caregiver cooking skills and later child outcomes may reflect developmental differences that had already emerged before fourth grade. In other words, caregiver cooking skills may contribute not only to subsequent changes during the two-year follow-up period but also to earlier developmental processes. The absence of significant interactions suggests that the associations between caregiver cooking skills and subsequent child resilience and prosocial behavior were broadly consistent across different baseline levels of these traits.

The mediation findings suggest that several family processes explain part of the association between caregiver cooking skills and child resilience. Family social capital showed the largest mediation proportion (22.4%), indicating that approximately one-fifth of the association may operate through stronger family trust, cohesion, and supportive relationships. Communication-related factors also contributed substantially, particularly talking about news with children (16.3%) and talking about school life (12.6%). In addition, healthy household routines, such as children’s vegetable intake (14.3%), jointly explained part of the association. These findings suggest that cooking skills may influence child resilience not through a single pathway, but through multiple interrelated family processes. The remaining association may reflect direct effects of cooking skills or other unmeasured pathways, such as overall diet quality, parenting practices, or children’s own self-regulation.

To the best of our knowledge, this is the first study to show the association between caregiver cooking skills and adolescent resilience. We found that food-related household routines partially mediated the association between caregiver cooking skills and child resilience. This is consistent with the results for frequency of home cooking and child resilience, which were mediated by child vegetable intake [23]. When caregivers had higher cooking skills, their child consumed more vegetables and cooked with their caregivers more often (Table 1). Therefore, higher caregiver cooking skills may not only ensure the quality of the children’s diet but also help children acquire self-care skills, such as making healthy food choices and preparing meals, which may contribute to higher resilience. Furthermore, the novelty of this study is that we identified a potentially modifiable factor associated with positive mental health in adolescents. From a developmental perspective, these food-related household routines may function as repeated proximal processes through which children acquire autonomy, problem-solving skills, and confidence in everyday life. Thus, caregiver cooking skills may contribute to resilience not only through nutritional pathways but also by fostering adaptive competencies through daily practice.

Caregiver-child interactions and family social capital mediated the associations between caregiver cooking skills and child resilience and prosocial behaviors. From a family systems perspective, cooking skills may be viewed as a household resource that shapes the quality of everyday family interactions, particularly during meal preparation and shared mealtimes. In this context, family social capital reflects relational resources within the household, including trust, communication, cohesion, and mutual support. This is consistent with the results for frequency of home cooking and child resilience and prosocial behaviors, which were mediated by parent-child interaction [23]. A survey of 10–14 years old in Japan found that they spent only 2–3 h with their parents on weekdays, and meals were the main activity they spent time together [28]. We found that caregivers with higher cooking skills engage in more frequent conversations with children compared to those with lower cooking skills. Japan has a cultural word of “*Ofukurono-aji*” (“taste of mom’s home cooking”), which refers to a comfort food cooked by a mother, which reflects the traditional gender role that mothers are supposed to cook at home, but for now, this means “taste of home” as father or grandparents can be the main chef at home. The high level of caregiver cooking skills enables them to prepare meals with the family’s physical and mental health in mind. Furthermore, eating together allows for observing the children’s condition and has the potential to strengthen family cohesion through “taste of home.”

In addition, the high level of caregiver cooking skills may increase their own resilience and prosocial behaviors, and may serve as role models for their children. A cross-sectional study conducted with a small convenience sample of adults in the U.S. reported that adults with higher cooking skills had higher resilience [44]. Cooking meals for the family is itself a prosocial behavior. Therefore, a high level of caregiver cooking skills may be linked to higher adolescent resilience and prosocial behaviors by demonstrating prosocial behaviors through cooking for family members and providing a sense of being cared for through cooked meals. The mediating role of family social capital suggests that the influence of caregiver cooking skills is not limited to food provision itself, but extends to strengthening family relationships that support child development. These findings are consistent with ecological models of development, which emphasize that repeated supportive interactions within the home environment are central to socioemotional growth.

We found that caregiver cooking skills were associated with greater increases in resilience among children in grades 4 through 6. This period is marked by increased stress in both friendships and academics, that is, children began to spend more time with friends or activities outside of school [45]. For example, in Japan, based on a nationwide sample, the prevalence of children attending cram schools late into the evening increases starting in grade 4, exceeding 30% by grade 6, reducing the time spent with caregivers after school [46]. Caregiver cooking skills that could enhance caregiver-child interaction during this period may have contributed to further resilience development.

Moreover, caregiver cooking skills were associated with higher levels of children’s prosocial behavior at follow-up, after adjusting for baseline levels. This may suggest a potential buffering effect on the age-related decline in prosocial behavior observed during this developmental period. Adolescence is a period when the motivation for prosocial behavior shifts from extrinsic motives, such as seeking praise from others, to intrinsic motives rooted in consideration for others, leading to a decrease in prosocial behavior [47]. One possible interpretation is that children’s appreciation of caregivers’ cooking may contribute to such behaviors. This may be understood in the context of social learning theory, which suggests that caregivers’ behaviors can serve as role models for children [17]. Therefore, when caregivers have high cooking skills, children may be more likely to recognize the value of intrinsic prosocial behavior and maintain high levels of it. This interpretation is consistent with social learning theory, whereby children internalize prosocial norms through observing repeated caregiving behaviors directed toward other family members.

This study had several limitations. First, only one caregiver in the family was able to assess the cooking skills. More than 90% of the caregivers whose cooking skills could be evaluated in this study were mothers. In most Japanese families with school-age children, the mother is in charge of meal preparation [48], suggesting that the influence of cooking skills of non-mothers on the children is considered to be small. Second, the quality of the meals prepared at home was not assessed. A group of caregivers’ high cooking skills may include those who prepare low-quality meals. However, we confirmed that higher caregiver cooking skills were associated with higher child vegetable intake. Third, common method bias and social desirability bias may have occurred. This is because caregivers’ cooking skills and children’s mental health were assessed using caregiver-reported questionnaires. However, caregiver cooking skills were not associated with maternal educational attainment (Table 1), suggesting that social desirability bias may be limited. In addition, the anonymous survey design may have helped reduce socially desirable responding. To address this common source bias, it is necessary to either collect information from third parties, such as teachers who can assess children’s mental health. Fourth, residual confounding and alternative explanations should be considered. Although the longitudinal design and adjustment for multiple socioeconomic and caregiver characteristics strengthen temporal inference, caregiver cooking skills may reflect broader family attributes rather than an isolated causal factor. For example, higher cooking skills may be a marker of caregiver conscientiousness, time availability, planning ability, or household organization, all of which could independently support children’s socioemotional development. In addition, unmeasured family characteristics may simultaneously promote both caregiver cooking skills and positive child outcomes. Such characteristics may also include caregivers’ own childhood experiences, such as exposure to maltreatment, which may influence later parenting practices as well as child socioemotional development. Selection effects may also contribute to the findings, as families who place a higher value on family cohesion, health, or child development may be more likely to invest in cooking practices as well as in behaviors that foster children’s socioemotional development. In this sense, caregiver cooking skills may partly reflect underlying family values rather than acting as an independent causal factor. Fifth, because follow-up data were collected in October 2020 during the COVID-19 pandemic, family routines, parental work patterns, and children’s psychosocial outcomes may have been influenced by pandemic-related circumstances. Therefore, the observed associations should be interpreted with caution. Finally, since this study was conducted only on students in public schools in one city in Tokyo, the generalizability may not be as high for adolescents living in other regions of Japan or attending private schools.

Based on our findings, we recommend launching cooking classes for caregivers and educational programs for those preparing to become parents. For example, iCook 4-H is an after-school program for youth ages 9 and 10, and the adults responsible for preparing meals [49]. Following the intervention, improvements in parental cooking skills were observed. In addition, family bonding, such as increased engagement of all family members in mealtime conversation, was also increased [50]. This program was designed to prevent childhood obesity; however, further research should focus on how a cooking skills intervention targeting parents of early adolescents affects children’s resilience and altruistic behaviors.

This longitudinal study provided new insights into the association between caregiver cooking skills and adolescent resilience and prosocial behavior. Higher caregiver cooking skills were associated with higher resilience and prosocial behavior in early adolescents aged 11–12 in Japan. Food-related household routines, caregiver-child interactions, and family cohesion mediated these associations. Creating an educational program that allows caregivers to learn cooking skills may be important to the positive mental health of early adolescents.

## Supplementary Information

Supplementary Material 1.

## Data Availability

Due to ethical restrictions, the data used to support the findings of this study cannot be made publicly available. However, they are available from the corresponding author upon reasonable request.
