# Supplementary material for: Do caregiver cooking skills boost adolescent resilience and prosocial behavior? Results from a population-based longitudinal study in Japan
Source: BMC Psychol. 2026 May 1;14:891. doi: 10.1186/s40359-026-04658-4 (PMC13277165; doi:10.1186/s40359-026-04658-4)
Supplement: Supplementary file 1 — Supplementary Material 1. [file 40359_2026_4658_MOESM1_ESM.docx]

The baseline survey was conducted in 2018 (n=5,311)

The participants who responded

baseline questionnaires

(n=4,605, response rate: 87%)

Disagreement or invalid response (n=315)

Eligible participants at baseline (n=4,290)

Non-response (n=557)

The participants who completed follow-up questionnaires in 2020 (grade 6)

(n=3,733, follow-up rate: 87%)

Excluded participants

Incomplete data on child sex (n = 7)

Missing data on child resilience (n = 7)

Missing data on child prosocial behavior (n = 78)

Analyzed participants (n=3,641)

**Supplementary Figure 1.** The participants flow for the analytic sample.

Food-related household routines:

Child’s vegetable intake

Child’s breakfast intake

Eating dinner with caregivers and children

Caregiver’s home cooking

Cooking with caregiver and child

Going out with caregiver and child

Caregiver-child interactions:

Talking about school life with caregiver and child

Talking about news with caregiver and child

Talking about TV shows with caregiver and child

Family cohesion:

Family social capital

Child’s

Resilience/Prosocial behavior

Caregiver’s

Cooking skills

**Supplementary Figure 2.** Hypothesis for the association between caregiver cooking skills and child resilience/prosocial behavior.

**Supplementary Table 1.** Summary of children’s resilience and prosocial behaviors scores at baseline (grade 4) and follow-up (grade 6) and their Spearman correlation coefficients (n=3,641).

|  |  | Mean | SD | Median | 1 | 2 | 3 | 4 |
| --- | --- | --- | --- | --- | --- | --- | --- | --- |
| 1 | Resilience at baseline (CRCS score: 0-100) | 69.6 | 16.3 | 71.9 | 1.00 |  |  |  |
| 2 | Resilience at follow-up (CRCS score: 0-100) | 71.7 | 16.3 | 70.0 | **0.64** | 1.00 |  |  |
| 3 | Prosocial behaviors at baseline (SDQ score: 0-100) | 67.3 | 20.8 | 71.9 | **0.45** | **0.36** | 1.00 |  |
| 4 | Prosocial behaviors at follow-up (SDQ score: 0-100) | 65.5 | 21.3 | 70.0 | **0.34** | **0.44** | **0.55** | 1.00 |

SD = standard deviation.

*Bold shows significance at p < 0.05.

**Supplementary Table 2.** Child resilience and prosocial behavior scores at follow-up (grade 6) according to caregiver cooking skills (n=3,641).

| Caregiver's cooking skills | N | Resilience score at follow-up (CRCS, 0–100) | Prosocial behavior score at follow-up (SDQ, 0–100) |
| --- | --- | --- | --- |
|  |  | Mean (SD) | Mean (SD) |
| Q1 (Low) | 1,160 | 66.7 (16.9) | 60.5 (21.6) |
| Q2 | 893 | 71.5 (15.5) | 65.3 (20.9) |
| Q3 | 696 | 74.0 (14.7) | 67.4 (20.2) |
| Q4 (High) | 892 | 76.5 (15.6) | 70.8 (20.8) |

CRCS = Children's Resilient Coping Scale; SD=Standard Deviation; SDQ = Strengths and Difficulties Questionnaire.

**Supplementary Figure 3.** Associations between caregiver cooking skills and children’s resilience at 2-year follow-up, including interaction terms with baseline resilience.

|  |  | Model 1 | Model 2 |
| --- | --- | --- | --- |
|  |  | coefficient (95%CI) | coefficient (95%CI) |
| Caregiver's cooking skills | |  |  |
|  | Q1 (Low) | ref | ref |
|  | Q2 | **4.06 (2.69 to 5.44)** | **2.95 (0.98 to 4.92)** |
|  | Q3 | **5.89 (4.40 to 7.37)** | **3.48 (1.06 to 5.91)** |
|  | Q4 (High) | **8.75 (7.38 to 10.1)** | **4.28 (1.97 to 6.60)** |
| Child's resilience at baseline (CRCS score, Tertile) | |  |  |
|  | T1 (Low) |  | ref |
|  | T2 (Middle) |  | **12.8 (11.0 to 14.7)** |
|  | T3 (High) |  | **22.9 (20.5 to 25.2)** |
| Caregiver's cooking skills x Child's resilience at baseline | |  |  |
|  | Caregiver's cooking skills Q2 & baseline child resilience T2 (Middle) |  | -1.41 (-4.19 to 1.38) |
|  | Caregiver's cooking skills Q2 & baseline child resilience T3 (High) |  | 0.04 (-3.30 to 3.38) |
|  | Caregiver's cooking skills Q3 & baseline child resilience T2 (Middle) |  | -2.30 (-5.50 to 0.91) |
|  | Caregiver's cooking skills Q3 & baseline child resilience T3 (High) |  | -1.50 (-5.10 to 2.10) |
|  | Caregiver's cooking skills Q4 & baseline child resilience T2 (Middle) |  | -1.13 (-4.22 to 1.96) |
|  | Caregiver's cooking skills Q4 & baseline child resilience T3 (High) |  | -0.76 (-4.13 to 2.60) |

CI = confidence interval; CRCS = Children's Resilient Coping Scale.

Coefficients represent mean differences in children’s outcome scores (range 0–100), with higher scores indicating higher levels of resilience.

Model 1: Adjusted for child's sex, cohabitation status, household income, respondent, respondent's K6, mother's employment status, father's age, education, and employment status.

Model 2: Model 1 + adjusted for baseline child's resilience and interaction term between caregiver cooking skills and child's resilience at baseline.

**Supplementary Figure 4.** Associations between caregiver cooking skills and children’s prosocial behavior at 2-year follow-up, including interaction terms with baseline prosocial behavior.

|  |  | Model 1 | Model 2 |
| --- | --- | --- | --- |
|  |  | coefficient (95%CI) | coefficient (95%CI) |
| Caregiver's cooking skills | |  |  |
|  | Q1 (Low) | ref | ref |
|  | Q2 | **4.06 (2.69 to 5.44)** | **3.78 (1.50 to 6.05)** |
|  | Q3 | **5.89 (4.40 to 7.37)** | **4.58 (1.95 to 7.22)** |
|  | Q4 (High) | **8.75 (7.38 to 10.1)** | **5.54 (2.95 to 8.13)** |
| Child's prosocial behavior at baseline (SDQ score, Tertile) | |  |  |
|  | T1 (Low) |  | ref |
|  | T2 (Middle) |  | **14.3 (11.8 to 16.8)** |
|  | T3 (High) |  | **27.2 (24.1 to 30.2)** |
| Caregiver's cooking skills x Child's prosocial behavior at baseline | |  |  |
|  | Caregiver's cooking skills Q2 & baseline child prosocial behavior T2 (Middle) |  | -1.38 (-5.16 to 2.40) |
|  | Caregiver's cooking skills Q2 & baseline child prosocial behavior T3 (High) |  | -1.42 (-5.83 to 2.98) |
|  | Caregiver's cooking skills Q3 & baseline child prosocial behavior T2 (Middle) |  | -2.20 (-6.25 to 1.85) |
|  | Caregiver's cooking skills Q3 & baseline child prosocial behavior T3 (High) |  | -3.23 (-7.90 to 1.44) |
|  | Caregiver's cooking skills Q4 & baseline child prosocial behavior T2 (Middle) |  | -0.58 (-4.47 to 3.31) |
|  | Caregiver's cooking skills Q4 & baseline child prosocial behavior T3 (High) |  | -3.89 (-8.18 to 0.40) |

CI = confidence interval; SDQ = Strengths and Difficulties Questionnaire.

Coefficients represent mean differences in children’s outcome scores (range 0–100), with higher scores indicating higher levels of prosocial behavior.

Model 1: Adjusted for child's sex, cohabitation status, household income, respondent, respondent's K6, mother's employment status, father's age, education, and employment status.

Model 2: Model 1 + adjusted for baseline child's prosocial behavior and interaction term between caregiver cooking skills and child’s prosocial behavior at baseline.

**Supplementary Table 5.** Associations between caregiver cooking skills and child resilience/prosocial behaviors according to household income level.

|  | Household income (million yen) | Caregiver's cooking skills | Crude | Model 1 |
| --- | --- | --- | --- | --- |
|  |  |  | coefficient (95%CI) | coefficient (95%CI) |
| Resilience (CRCS score) | | |  |  |
|  | <3.00 (n=354) | Q1 (Low) | ref | ref |
|  |  | Q2 | 4.36 (-1.06 to 9.79) | 4.64 (-0.82 to 10.1) |
|  |  | Q3 | 4.36 (-1.06 to 9.79) | 4.64 (-0.82 to 10.1) |
|  |  | Q4 (High) | **7.67 (3.41 to 11.9)** | **7.77 (3.46 to 12.1)** |
|  | 3.00–5.99 (n=1,083) | Q1 (Low) | ref | ref |
|  |  | Q2 | **6.91 (4.82 to 9.01)** | **6.24 (4.12 to 8.37)** |
|  |  | Q3 | **6.91 (4.82 to 9.01)** | **6.24 (4.12 to 8.37)** |
|  |  | Q4 (High) | **11.64 (9.08 to 14.2)** | **10.23 (7.66 to 12.8)** |
|  | 6.00-9.99 (n=1,256) | Q1 (Low) | ref | ref |
|  |  | Q2 | **4.94 (2.77 to 7.12)** | **4.23 (2.05 to 6.41)** |
|  |  | Q3 | **4.94 (2.77 to 7.12)** | **4.23 (2.05 to 6.41)** |
|  |  | Q4 (High) | **6.94 (4.87 to 9.01)** | **6.61 (4.55 to 8.67)** |
|  | ≥10.0 (n=455) | Q1 (Low) | ref | ref |
|  |  | Q2 | 2.36 (-1.45 to 6.17) | 1.41 (-2.41 to 5.23) |
|  |  | Q3 | 2.36 (-1.45 to 6.17) | 1.41 (-2.41 to 5.23) |
|  |  | Q4 (High) | **4.73 (1.24 to 8.22)** | 3.49 (-0.04 to 7.03) |
| Prosocial behavior (SDQ score) | | |  |  |
|  | <3.00 (n=354) | Q1 (Low) | ref | ref |
|  |  | Q2 | **9.79 (3.46 to 16.1)** | **9.74 (3.30 to 16.2)** |
|  |  | Q3 | **9.18 (1.94 to 16.4)** | **8.01 (0.63 to 15.4)** |
|  |  | Q4 (High) | **10.87 (4.95 to 16.8)** | **10.26 (4.21 to 16.3)** |
|  | 3.00–5.99 (n=1,083) | Q1 (Low) | ref | ref |
|  |  | Q2 | **3.52 (0.33 to 6.70)** | **3.63 (0.42 to 6.83)** |
|  |  | Q3 | **9.71 (6.11 to 13.3)** | **8.88 (5.23 to 12.5)** |
|  |  | Q4 (High) | **10.25 (6.84 to 13.7)** | **9.18 (5.75 to 12.6)** |
|  | 6.00-9.99 (n=1,256) | Q1 (Low) | ref | ref |
|  |  | Q2 | **3.23 (0.04 to 6.4)** | **3.76 (0.54 to 6.98)** |
|  |  | Q3 | **4.53 (1.23 to 7.8)** | **4.93 (1.58 to 8.29)** |
|  |  | Q4 (High) | **9.84 (6.66 to 13.0)** | **9.39 (6.21 to 12.6)** |
|  | ≥10.0 (n=455) | Q1 (Low) | ref | ref |
|  |  | Q2 | **9.86 (4.61 to 15.1)** | **9.05 (3.63 to 14.5)** |
|  |  | Q3 | **8.48 (2.96 to 14.0)** | **6.88 (1.18 to 12.6)** |
|  |  | Q4 (High) | **8.34 (3.20 to 13.5)** | **6.39 (1.04 to 11.8)** |

CI = confidence interval; CRCS = Children’s Resilient Coping Scale; SD = Standard Deviation; SDQ = Strengths and Difficulties Questionnaire.

Coefficients represent mean differences in follow-up resilience or prosocial behavior scores compared with Q1 (lowest caregiver cooking skills), estimated using linear regression models.

Outcome scores range from 0 to 100, with higher scores indicating higher levels of resilience or prosocial behavior.

Model 1: Adjusted for child's sex, cohabitation status, respondent, respondent's K6, mother's employment status, father's age, education, and employment status.

**Supplementary Table 6.** Associations between caregiver cooking skills and child resilience/prosocial behaviors according to child's sex.

|  | Child's sex | Caregiver's cooking skills | Crude | Model 1 |
| --- | --- | --- | --- | --- |
|  |  |  | coefficient (95%CI) | coefficient (95%CI) |
| Resilience (CRCS score) | | |  |  |
|  | Boy (n=1,821) | Q1 (Low) | ref | ref |
|  |  | Q2 | **3.44 (1.50 to 5.38)** | **2.70 (0.75 to 4.64)** |
|  |  | Q3 | **6.71 (4.55 to 8.87)** | **5.42 (3.25 to 7.60)** |
|  |  | Q4 (High) | **9.37 (7.36 to 11.4)** | **8.57 (6.56 to 10.6)** |
|  | Girl (n=1,820) | Q1 (Low) | ref | ref |
|  |  | Q2 | **6.57 (4.62 to 8.52)** | **5.71 (3.74 to 7.67)** |
|  |  | Q3 | **7.71 (5.68 to 9.74)** | **6.56 (4.51 to 8.60)** |
|  |  | Q4 (High) | **9.93 (8.04 to 11.8)** | **8.98 (7.09 to 10.9)** |
| Prosocial behavior (SDQ score) | | |  |  |
|  | Boy (n=1,821) | Q1 (Low) | ref | ref |
|  |  | Q2 | **3.57 (1.02 to 6.1)** | **3.46 (0.88 to 6.04)** |
|  |  | Q3 | **6.60 (3.76 to 9.4)** | **6.29 (3.40 to 9.17)** |
|  |  | Q4 (High) | **10.6 (7.93 to 13.2)** | **9.98 (7.32 to 12.6)** |
|  | Girl (n=1,820) | Q1 (Low) | ref | ref |
|  |  | Q2 | **6.39 (3.81 to 9.0)** | **5.76 (3.12 to 8.40)** |
|  |  | Q3 | **6.77 (4.09 to 9.5)** | **6.03 (3.29 to 8.77)** |
|  |  | Q4 (High) | **9.6 (7.09 to 12.1)** | **9.07 (6.53 to 11.6)** |

CI = confidence interval; CRCS = Children’s Resilient Coping Scale; SD = Standard Deviation; SDQ = Strengths and Difficulties Questionnaire.

Coefficients represent mean differences in follow-up resilience or prosocial behavior scores compared with Q1 (lowest caregiver cooking skills), estimated using linear regression models.

Outcome scores range from 0 to 100, with higher scores indicating higher levels of resilience or prosocial behavior.

Model 1: Adjusted for child's sex, cohabitation status, respondent, respondent's K6, mother's employment status, father's age, education, and employment status.

**Supplementary Table 7.** Associations between caregiver cooking skills and child resilience/prosocial behaviors among mother respondents only (n=3,332).

|  | Caregiver's cooking skills | Crude | Model 1 |
| --- | --- | --- | --- |
|  |  | coefficient (95%CI) | coefficient (95%CI) |
| Resilience (CRCS score) | |  |  |
|  | Q1 (Low) | ref | ref |
|  | Q2 | **4.61 (3.17 to 6.04)** | **4.04 (2.63 to 5.46)** |
|  | Q3 | **6.83 (5.28 to 8.37)** | **5.64 (4.12 to 7.17)** |
|  | Q4 (High) | **9.26 (7.80 to 10.7)** | **8.24 (6.79 to 9.7)** |
| Prosocial behavior (SDQ score) | | |  |
|  | Q1 (Low) | ref | ref |
|  | Q2 | **4.76 (2.84 to 6.7)** | **4.71 (2.80 to 6.61)** |
|  | Q3 | **6.54 (4.48 to 8.6)** | **5.91 (3.86 to 7.96)** |
|  | Q4 (High) | **10.5 (8.51 to 12.4)** | **9.48 (7.54 to 11.4)** |

CI = confidence interval; CRCS = Children’s Resilient Coping Scale; SD = Standard Deviation; SDQ = Strengths and Difficulties Questionnaire.

Coefficients represent mean differences in follow-up resilience or prosocial behavior scores compared with Q1 (lowest caregiver cooking skills), estimated using linear regression models.

Outcome scores range from 0 to 100, with higher scores indicating higher levels of resilience or prosocial behavior.

Model 1: Adjusted for child's sex, cohabitation status, respondent, respondent's K6, mother's employment status, father's age, education, and employment status.

**Supplementary Table 8.** Associations between caregiver cooking skills and child resilience/prosocial behaviors under alternative covariate adjustment models.

|  | Caregiver's cooking skills | Crude | Model 1 | Model 2 | Model 3 |
| --- | --- | --- | --- | --- | --- |
|  |  | coefficient (95%CI) | coefficient (95%CI) | coefficient (95%CI) | coefficient (95%CI) |
| Resilience (CRCS score) | | |  |  |  |
|  | Q1 (Low) | ref | ref | ref | ref |
|  | Q2 | **7.33 (5.84 to 8.82)** | **4.92 (3.55 to 6.30)** | **4.84 (3.47 to 6.21)** | **4.06 (2.69 to 5.44)** |
|  | Q3 | **9.77 (8.39 to 11.15)** | **7.20 (5.71 to 8.68)** | **6.94 (5.47 to 8.42)** | **5.89 (4.40 to 7.37)** |
|  | Q4 (High) | **0.00 (0.00 to 0.00)** | **9.62 (8.24 to 11.0)** | **9.53 (8.16 to 10.9)** | **8.75 (7.38 to 10.12)** |
| Prosocial behavior (SDQ score) | | |  |  |  |
|  | Q1 (Low) | ref | ref | ref | ref |
|  | Q2 | **4.76 (2.93 to 6.59)** | **4.89 (3.08 to 6.7)** | **4.99 (3.18 to 6.80)** | **4.72 (2.89 to 6.56)** |
|  | Q3 | **6.90 (4.93 to 8.87)** | **6.67 (4.72 to 8.6)** | **6.83 (4.87 to 8.78)** | **6.31 (4.32 to 8.29)** |
|  | Q4 (High) | **10.28 (8.45 to 12.12)** | **10.0 (8.22 to 11.8)** | **10.00 (8.18 to 11.8)** | **9.51 (7.68 to 11.34)** |

CI = confidence interval; CRCS = Children’s Resilient Coping Scale; SD = Standard Deviation; SDQ = Strengths and Difficulties Questionnaire.

Coefficients represent mean differences in follow-up resilience or prosocial behavior scores compared with Q1 (lowest caregiver cooking skills), estimated using linear regression models.

Outcome scores range from 0 to 100, with higher scores indicating higher levels of resilience or prosocial behavior.

Model 1: Adjusted for child's sex.

Model 2: Model 1 + adjusted for household status (cohabitation status, household income).

Model 3: Model 2 + adjusted for caregiver's status (respondent, respondent's K6, mother's employment status, father's age, education, and employment status).
